# Supplementary material for: Effect of two-dimensional spatial confinement on platelet mechanics
Source: Biophys Rep (N Y). 2026 Apr 10;6(2):100261. doi: 10.1016/j.bpr.2026.100261 (PMC13136703; doi:10.1016/j.bpr.2026.100261)
Supplement: Document S1. Figures S1–S7 [file mmc1.pdf]

Biophysical Reports, Volume *6*

Supplemental information

Effect of two-dimensional spatial confinement on platelet mechanics

Aylin Balmes, Vincent Gidlund, and Tilman E. Schäffer

Supplemental Information

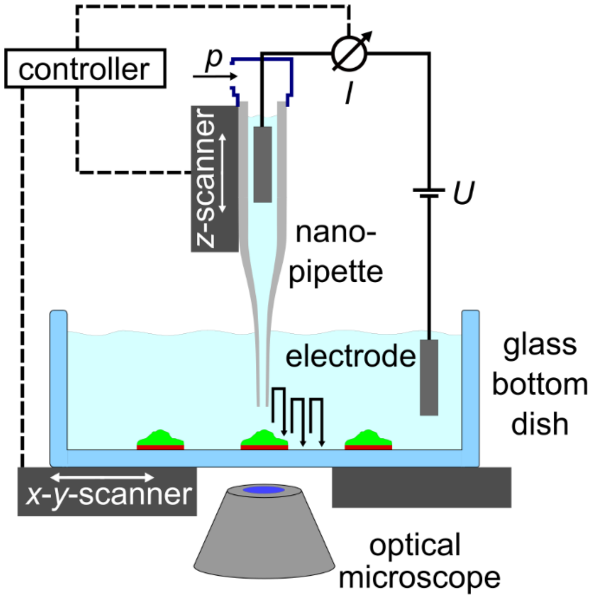

Figure S1: Principle of scanning ion conductance microscopy (SICM). A voltage (*U*) applied between two electrodes - one in the electrolyte-filled nanopipette and the other in the bath - induces an ion current (*I*) that depends on the pipette-sample distance. The topography of the sample is imaged by scanning it using an *x*-*y*-scanner. At every *x*-*y*-position, the nanopipette is vertically approached toward the sample until the ion current drops to a predetermined threshold, at which point the corresponding *z*-position of the nanopipette is recorded. The recorded *z*-positions make up the topography image. For stiffness imaging, a pressure (*p*) is applied to the top end of the nanopipette. When approaching the pipette to a soft sample, it is deformed by the outward flow from the pipette, resulting in a shallower slope of the ion current vs. distance curve than for a stiff sample. Stiffness is derived from this slope [1].

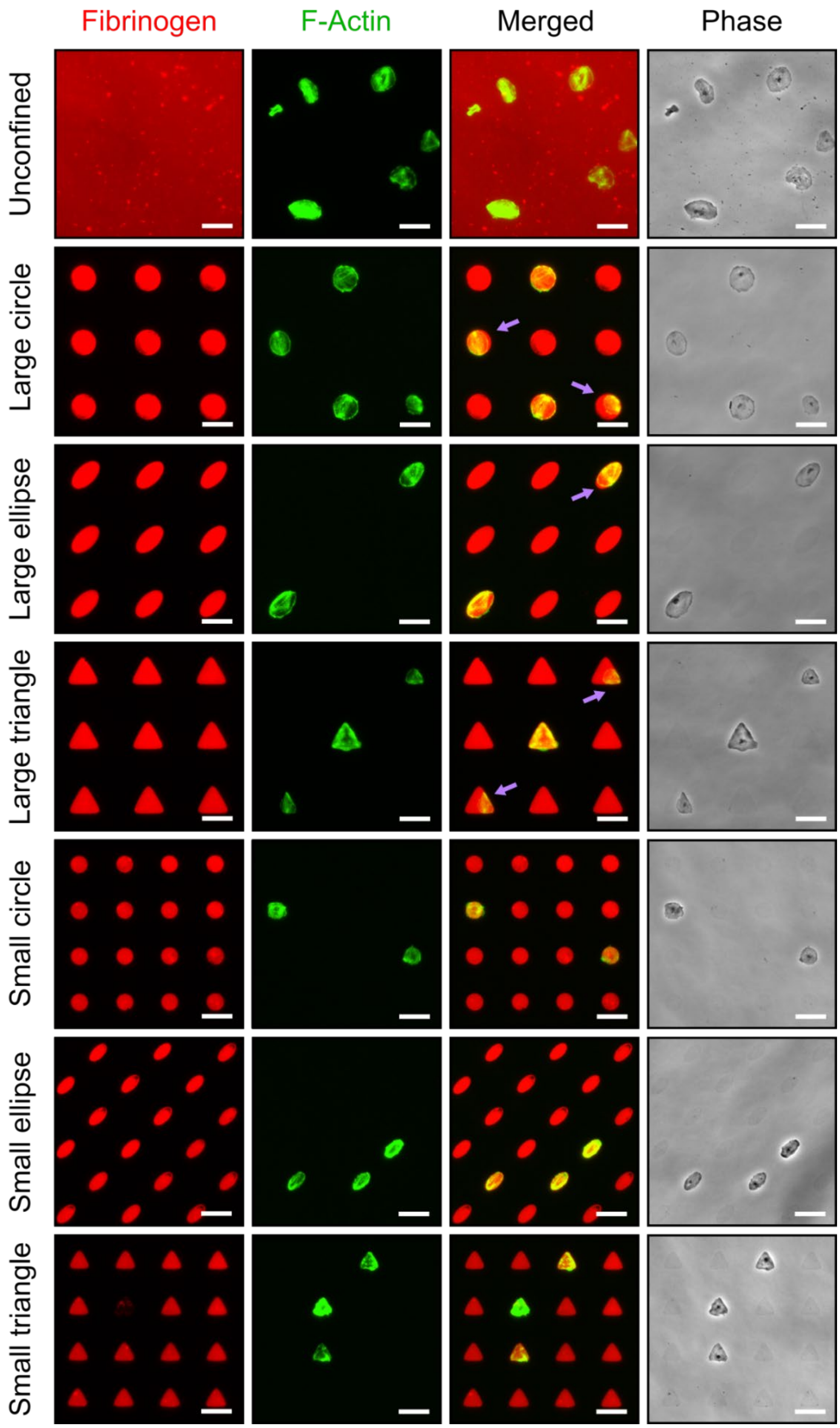

Figure S2: Overview of fibrinogen micropatterns and spatially confined platelets. Fibrinogen (red) and F-actin (green) fluorescence with merged and phase contrast views of platelets on fibrinogen micropatterns of varying shape and size. Light purple arrows indicate platelets that do not fully cover the micropatterns. Scale bars: 10 µm.

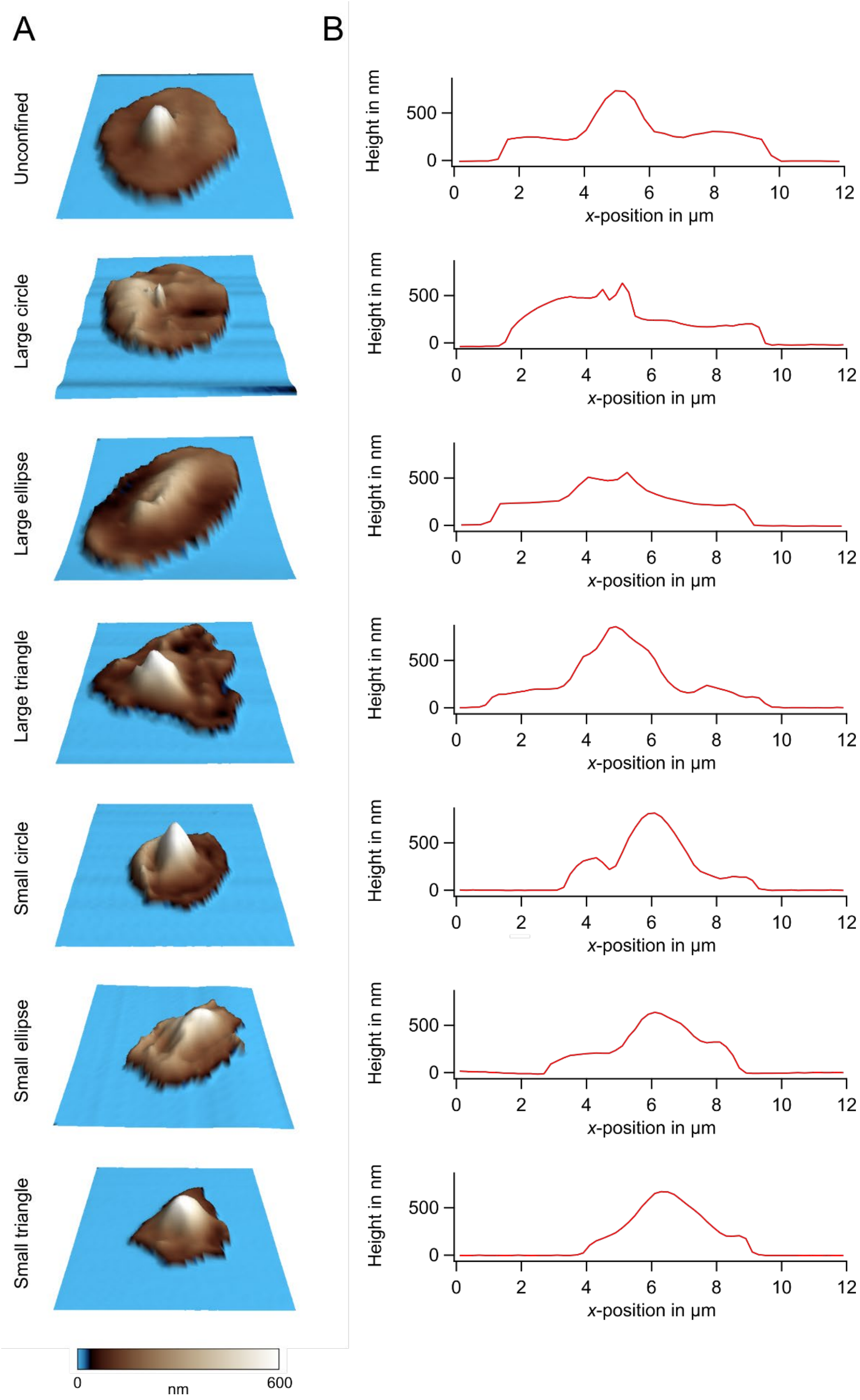

Figure S3: Visualization of three-dimensional platelet topography. (**A**) Three-dimensional renderings of platelet surface topography. The *x*-*y*-image size is 12 × 12 µm². (**B**) Respective height profiles in the horizontal (*x*) direction, taken through the point of maximum height.

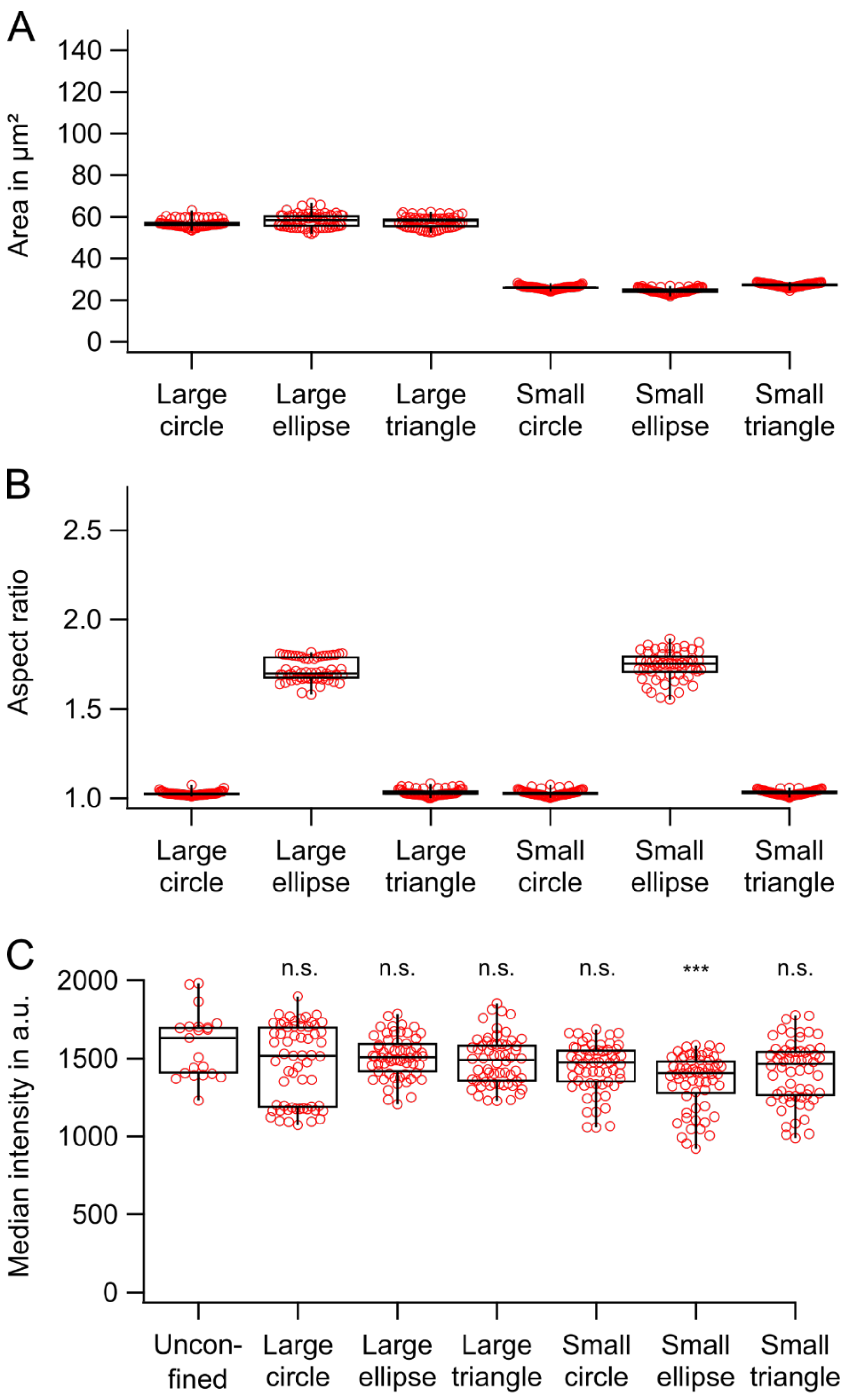

Figure S4: Fibrinogen micropatterns are highly uniform in shape and fluorescence. (**A**) Area and (**B**) aspect ratio are highly uniform. (**C**) The median fibrinogen fluorescence intensity of the micropatterns was not significantly different compared to flat printings (unconfined), except for the small ellipse micropatterns, where a slight, but significant decrease was observed (*n*=60 micropatterns, *n*=21 flat printings; Dunn's test).

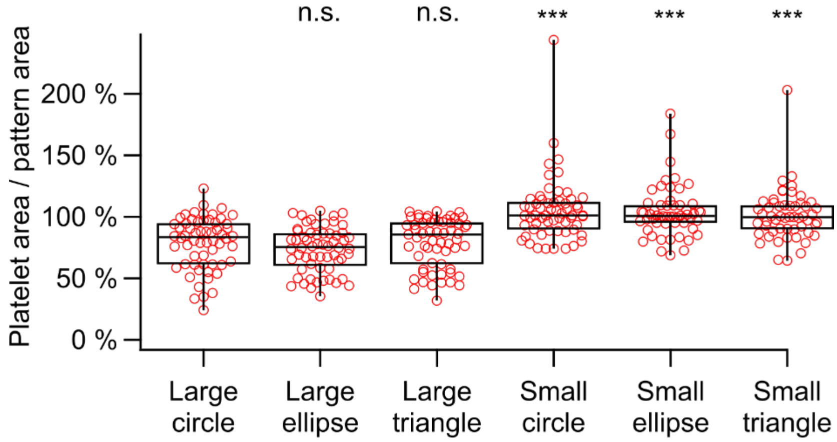

Figure S5: Ratios of platelet area to underlying pattern area are significantly higher for small patterns compared to large patterns. For each condition, n=54–62 platelets from 3–4 donors were analyzed. Significant differences between conditions are indicated by asterisks (*: $p < 0.05$; **: $p < 0.01$; ***: $p < 0.001$; n.s. indicates no significant difference; Dunn's test).

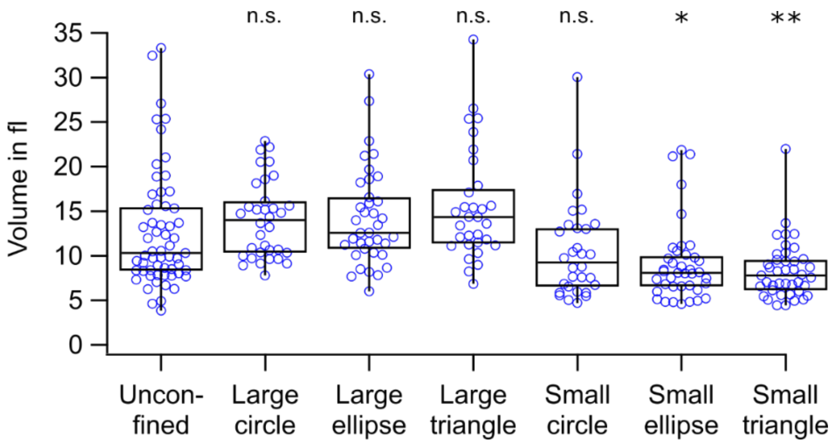

Figure S6: Platelet volume under spatial confinement. Volume was significantly reduced on small ellipse and small triangle micropatterns, compared to unconfined platelets (n=30–59 platelets per condition from 3–4 donors). Significant differences between unconfined condition and confined conditions are indicated by asterisks (*: $p < 0.05$; **: $p < 0.01$; ***: $p < 0.001$; n.s. indicates no significant difference; Dunn's test).

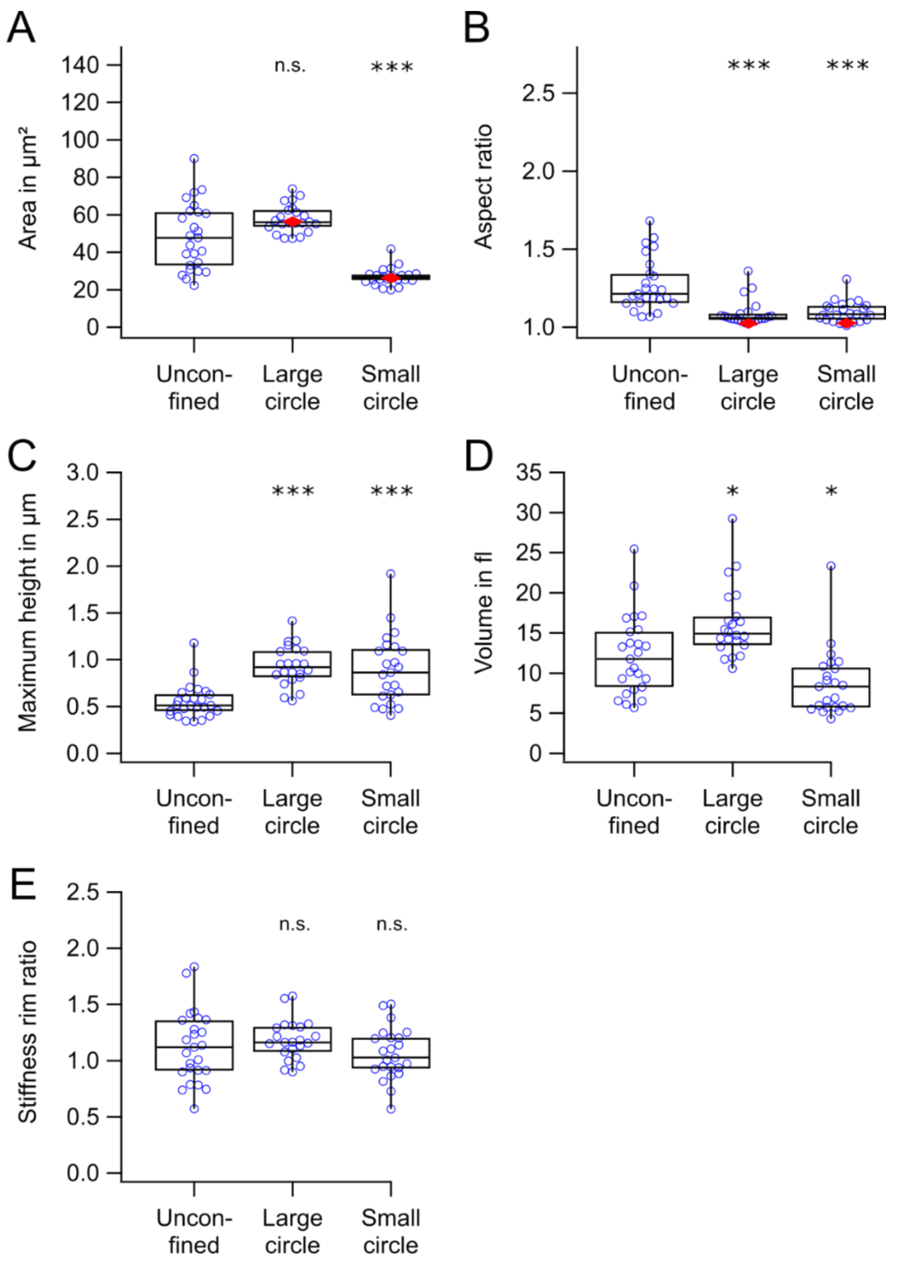

Figure S7: Topographical parameters and stiffness rim ratio of platelets treated with 8-Br-cGMP. (**A**) Platelet area was significantly reduced for platelets confined to small circle micropatterns, compared to unconfined platelets. Median areas of microcontact printed micropatterns are shown as red diamonds. (**B**) Platelet aspect ratio was significantly reduced for platelets confined to large or small circle micropatterns, compared to unconfined platelets. Median aspect ratios of microcontact-printed micropatterns are shown as red diamonds. (**C**) Platelet maximum height was significantly increased for platelets confined to large or small circle micropatterns, compared to unconfined platelets. (**D**) Platelet volume was significantly increased or reduced when confined to large or small circle micropatterns, respectively, compared to unconfined platelets. (**E**) Stiffness rim ratio was not significantly altered by spatial confinement. Each marker represents an individual platelet. Per condition, 22–25 platelets from 3 donors were measured. Significant differences between unconfined condition and confined conditions are indicated by asterisks (*: $p < 0.05$; **: $p < 0.01$; ***: $p < 0.001$; n.s. indicates no significant difference; Dunn's test).

References

1. Rheinlaender, J. and T.E. Schäffer, *Mapping the mechanical stiffness of live cells with the scanning ion conductance microscope.* Soft Matter, 2013. **9**(12): p. 3230–3236.
