# Supplementary material for: Effect of two-dimensional spatial confinement on platelet mechanics
Source: Biophys Rep (N Y). 2026 Apr 10;6(2):100261. doi: 10.1016/j.bpr.2026.100261 (PMC13136703; doi:10.1016/j.bpr.2026.100261)
Supplement: Document S2. Article plus supplemental information [file mmc2.pdf]

Biophysical Reports

ARTICLE

# Effect of two-dimensional spatial confinement on platelet mechanics

Aylin Balmes,[1,2] Vincent Gidlund,[1,2] and Tilman E. Schäffer[1,*]
[1]Institute of Applied Physics, University of Tübingen, Tübingen, Germany
[2]These authors contributed equally

ABSTRACT Platelets are small, anucleate cells critical for hemostasis and thrombosis. Within platelet plugs and narrow capillaries, they often encounter spatially confining microenvironments. To examine how confinement influences platelet properties, we employed microcontact printing to generate fibrinogen micropatterns of varying sizes and shapes. Platelets cultured on these micropatterns adapted both their morphology and mechanical characteristics. Scanning ion conductance microscopy revealed changes in area, aspect ratio, and height, while fluorescence microscopy showed F-actin redistribution toward the periphery. Confinement reduced platelet stiffness in a size-dependent but shape-independent manner. To explore the role of intracellular signaling, we examined cyclic guanosine monophosphate (cGMP), a key inhibitor of platelet activation, adhesion, and aggregation. Treatment with a cGMP analog preserved F-actin redistribution but prevented stiffness changes in response to confinement, indicating that cGMP inhibits stiffness modulation without affecting cytoskeletal reorganization.

WHY IT MATTERS Platelets are small blood cells that help stop bleeding by forming plugs in damaged blood vessels. Inside these plugs or in narrow vessels, platelets have limited space, but how they behave under such spatially confining environments is not fully understood. To study this, we used microcontact printing to create small protein patterns that spatially confine platelets. These patterns came in different sizes and shapes, such as circles, ellipses, and triangles. We found that platelets changed their shape to fit these patterns, and on small patterns they also alter their mechanical properties. Understanding how platelets respond to confining environments can improve our knowledge of plug formation and may lead to better treatments of blood-related diseases.

## INTRODUCTION

Platelets (thrombocytes) are key components of blood, playing essential roles in the initial inflammatory response,[1,2] blood coagulation, and hemostasis.[3,4] Upon vascular injury, platelets activate, adhere to extracellular matrix proteins,[5] and undergo rapid morphological changes.[6,7] They release thrombin, ADP, and other mediators to recruit additional platelets, forming a plug that seals the injury.[8] The dysregulation of aggregation can cause thrombotic diseases.[9]

Platelets often encounter spatially confining microenvironments within plugs or narrow capillaries. Confinement effects have been studied for decades.[10] Early results revealed a link between the size of two-dimensional (2D) confinement regions and cell apoptosis.[11] More recent research has shown considerable effects of confinement on cell migration.[12–14] Platelet behavior in one-dimensional (1D)[15,16] and 2D[17] spatial confinement has been investigated using collagen or fibrinogen micropatterns. However, the impact of 2D confinement on platelet mechanics remains unclear.

Here, we created 2D fibrinogen micropatterns on glass surfaces to mimic confining microenvironments and analyzed platelet morphology and stiffness using scanning ion conductance microscopy (SICM) and fluorescence microscopy. SICM is a nanopipette-based imaging technique that has been used to measure platelet morphology[18,19] and stiffness.[20–23] SICM enables high-resolution imaging of live cells without physical contact between the nanopipette and the sample.[24,25] This noninvasive approach is particularly important for investigating platelets, as other scanning probe techniques, such as atomic

Submitted January 12, 2026, and accepted for publication April 8, 2026.
*Correspondence: tilman.schaeffer@uni-tuebingen.de
Editor: Erdinc Sezgin.
https://doi.org/10.1016/j.bpr.2026.100261
© 2026 The Author(s). Published by Elsevier Inc. on behalf of Biophysical Society.
This is an open access article under the CC BY license (http://creativecommons.org/licenses/by/4.0/).

force microscopy, have a higher risk of unintentionally activating[26] or damaging the measured platelets.

Using SICM, we found that platelets cultured on micropatterns of varying sizes and shapes (small and large circles, ellipses, and triangles) adapt their morphology to match these shapes. We also detected changes in platelet biomechanics, including a reduction in stiffness. Complementary fluorescence microscopy revealed a redistribution of F-actin.

To assess how platelet activation influences their behavior under spatial confinement, we inhibited activation using a membrane-permeable cyclic guanosine monophosphate (cGMP) analog. cGMP is a second messenger involved in numerous physiological processes.[27] In platelets, cGMP is synthesized by nitric oxide-sensitive guanylyl cyclase (NO-GC) and inhibits platelet activation, adhesion, and aggregation.[28–30] It also modulates platelet morphology and stiffness.[22] However, its effect on platelets within confining microenvironments has not yet been explored.

## MATERIALS AND METHODS

### Lithography

Patterns were designed using KLayout (https://github.com/KLayout/klayout). A 10-inch silicon wafer was spin-coated with SU8-3005 (30 s, 3,000 Rpm, acceleration 300 Rpm/s). The silicon wafer was placed on a preheated hot plate at 60°C for 60 s, followed by quickly heating the plate to 95°C and prebaking the wafer for another 180 s. The wafer was removed from the hot plate and left to cool down at room temperature for 3–5 min. It was then transferred to the maskless lithography setup (μMLA; Heidelberg Instruments, Heidelberg, Germany). Lithography was performed using optical focus mode, write mode I, and an exposure of 80 mJ/cm$^2$. For post-baking, the wafer was placed on a preheated hot plate at 60°C for 60 s before heating up the hot plate to 95°C, leaving the wafer there for another 120 s, then removing the wafer to cool it down at room temperature. The coated wafer was placed in a glass dish containing 1-methoxy-2-propanol acetate (PGMEA) and was placed on top of a vortexer (set to the lowest setting). After 60 s, the wafer was removed from the dish, rinsed with PGMEA, and transferred to another glass dish with PGMEA. After slowly vortexing for another 60 s, the wafer was removed from the dish, rinsed with PGMEA, and immediately dried using a nitrogen gun. An anti-adhesive coating was applied by pipetting 1–2 drops of trichloro(1*H*,1*H*,2*H*,2*H*-perfluorooctyl)silane into a heated (125°C) chamber containing the patterned wafer and a nitrogen atmosphere. After 30 min, the chamber was cooled to room temperature and the wafer was removed.

### Microcontact printing

Micropatterned glass-bottom substrates were prepared via microcontact printing. To create patterning stamps, viscous polydimethylsiloxane (PDMS) (Sylgard 184; Avantor, Radnor, PA, 10:1 monomer/crosslinker ratio) was poured over patterned silicon wafers, degassed for 30 min, and cured for 2 h at 80°C. A small piece of the cured PDMS elastomer was cut out and stored in a plastic dish with the patterned side facing upward until using it as a stamp. The stamp was washed once with 70% ethanol and dried under gentle nitrogen gas flow. After plasma cleaning the stamp for 30 s at 100% power (Zepto-QRS 200; Diener Electronic, Ebhausen, Germany), 100 μL of fibrinogen Alexa Fluor 594 (#F13193; Thermo Fisher Scientific, Waltham, MA) inking solution (57.7 μg/mL in Dulbecco's phosphate-buffered saline [PBS] [#D8537; Sigma-Aldrich, St. Louis, MO]) was applied before quickly covering the stamp with parafilm and incubating it for 30 min at 37°C. The parafilm was removed and the stamp washed once with deionized water, followed by drying under gentle nitrogen gas flow. The dried and inked stamp was placed on a plasma-cleaned (30 s, 100% power) glass-bottom dish (35 mm, #81218; ibidi, Graefelfing, Germany) with the patterned side facing downward. After incubation for 15 min at 37°C, the stamp was gently removed from the dish, leaving behind micropatterns mirroring the used stamp. The micropatterned dish was washed three times with deionized water. For storage, 1 mL of deionized water was added to the dish. The micropatterned dish was usable for several days.

### Surface passivation

To prevent platelet adhesion to nonpatterned regions, the micropatterned glass-bottom dishes were passivated by coating with 0.01 mg/mL poly(L-lysine)(20)-g[3.5]-poly(ethylene glycol)(2) (SuSoS, Duebendorf, Switzerland) in 10 mM HEPES (#H3537; Sigma-Aldrich) at 37°C for 1–3 h. The micropatterned, coated dishes were then washed three times with high-performance liquid chromatography water (#10449380; Fisher Scientific, Schwerte, Germany). For each measurement day, the coating was freshly prepared.

### Platelet isolation

All procedures were approved by the institutional ethics committee (273/2018BO2) and complied with the Declaration of Helsinki. Informed consent was obtained from all subjects involved in the study. Blood was withdrawn from healthy volunteers in acid-citrate dextrose (ACD) (#C3821, Sigma-Aldrich) (ACD/blood volume ratio 1:4) to avoid coagulation. Blood was stored at 37°C under continuous slow rotation until usage. Platelet-rich plasma (PRP) was obtained by centrifugation at 200 × *g* for 20 min. To obtain washed platelets, 1.5 mL of PRP were added to 4.5 mL of Tyrode buffer (137 mM NaCl, 3 mM KCl, 1 mM $MgCl_2$, 0.4 mM $NaH_2PO_4$, 12 mM $NaHCO_3$, 5.5 mM D-glucose, 4 mM HEPES, and 1 g/L bovine serum albumin) at pH 6.5, followed by a second centrifugation step at 880 × *g* for 10 min. The thereby formed platelet pellet was resuspended in Tyrode buffer (pH 7.4). The resulting platelet suspension was used for experiments immediately.

### Drug treatment

For all experiments, platelets were activated prior to seeding by incubation for 1 min with thrombin-supplemented Tyrode (pH 7.4) (0.13 U thrombin [#T6884; Sigma-Aldrich] per mL Tyrode, pH 7.4). Micropatterned glass-bottom dishes were incubated with 10 μL of platelet suspension for 10 min at 37°C and subsequently washed once with thrombin-supplemented Tyrode (pH 7.4) to remove nonadherent platelets. Platelets were allowed to spread for another 10 min at 37°C, and samples were then washed three times with thrombin-supplemented Tyrode (pH 7.4). The overall time between platelet seeding and the start of experiments was 30–45 min, including the time needed for washing and transferring the samples and setting up the microscope. For experiments with 8-bromo-cyclic guanosine monophosphate (8-Br-cGMP), the

platelet suspension was treated with 1 mM 8-Br-cGMP (#B1381; Sigma-Aldrich) for 10 min at 37°C prior to activation with thrombin-supplemented Tyrode (pH 7.4). The preparation protocol (i.e., amount of platelet suspension, adhesion, and spreading time) typically resulted in most micropatterns remaining unoccupied by platelets; however, this was necessary to prevent multiple platelets from adhering to the same micropattern.

## SICM imaging

SICM uses an electrolyte-filled nanopipette, over which a voltage is applied, to scan the sample (Figure S1). The ion current through the pipette, which depends on the pipette-sample distance, is measured, allowing for the recording of sample topography and mechanical properties. Two custom-built SICM setups described elsewhere[31,32] were used. Nanopipettes with an inner radius of about 90 nm were fabricated using a P-2000 $CO_2$-laser-based puller (Sutter Instruments, Novato, CA). A voltage of 200 mV and a pressure of 10 kPa were applied to the pipette for imaging. The ion current versus the vertical pipette position was recorded with a retract distance of 2 μm and an approach/retract velocity of 50 μm/s. Images were recorded with a scan size of $12 \times 12$ $\mu m^2$ or $16 \times 16$ $\mu m^2$ and a pixel size of 200 or 300 nm. The sample stiffness was determined from these current-position curves as previously described.[33,34] A multiplicative correction factor was applied to the measured stiffness values to compensate for the overestimation of stiffness of thin and soft samples on stiff substrates.[34]

## Fluorescence measurements

Immediately after drug treatment, the samples were fixed in PBS containing 2% formaldehyde (#F1635; Sigma-Aldrich) for 10 min. The samples were then washed three times with PBS and subsequently permeabilized in PBS containing 0.1% Triton X-100 (#X100, Sigma-Aldrich) for 10 min. After washing the samples three times with PBS again, they were stained using phalloidin iFluor 488 (#ab176753; Abcam, Cambridge, UK) diluted in PBS (volume ratio 1:2,000) for 20 min at room temperature in a dark humid chamber. After washing the samples three times with PBS, imaging was performed using an inverted optical microscope (Ti-E; Nikon, Tokyo, Japan) in wide-field and epifluorescence mode, using a 100× oil-immersion objective.

## Rim ratio determination

To determine the outline of platelets, binary masks were obtained from phase-contrast images using a convolutional neural network described elsewhere.[35] The F-actin rim ratio was defined as the arithmetic mean of F-actin fluorescence intensity in the rim region of a platelet divided by the arithmetic mean of F-actin fluorescence intensity in the central region of a platelet. To define the rim and central regions, first the effective radius $r_{eff}$ of the platelet was calculated from its area $A$ (determined from the binary mask) via $r_{eff} = \sqrt{A/\pi}$. The rim width $w_{rim}$ was chosen as $w_{rim} = \left(1 - \sqrt{\frac{1}{2}}\right) \times r_{eff}$. The central region was derived from the platelet mask by applying binary erosion with a structuring element of size $w_{rim}$. The rim region was defined as the platelet mask excluding the central region. Typically, the central region accounted for about one-third of the platelet area $A$, while the rim region comprised the remaining two-thirds.

For calculating height and stiffness rim ratios, platelet binary masks were generated using a height threshold of 50 nm. The rim and central regions were defined analogously as for the F-actin rim ratio determination. The height rim ratio (stiffness rim ratio) was calculated as the ratio between the arithmetic mean height (geometric mean stiffness) in the rim region and the arithmetic mean height (geometric mean stiffness) in the central region. For determination of the rim ratios, only platelets that fully overlapped with the micropattern were considered.

## Statistics

Data analysis was performed in Igor Pro (Wavemetrics, Portland, OR). Boxplots show the median and the lower and upper quartiles. Data were tested for normality using the Shapiro-Wilk test. As the data were typically not normally distributed, they were tested using the Dunn's test (for comparison of three groups or more) or the Wilcoxon-Mann-Whitney test (for comparison of two groups). The Dunn's test is a nonparametric, multicomparison test for multiple groups with different sample sizes. Results were considered significant for $p$-values of <0.05. Nonsignificant results are indicated as n.s., with asterisks indicating $p$-values as $p < 0.001$, $p < 0.01$, and $p < 0.05$ in all figures.

# RESULTS

## Platelets adapted to spatial confinement

Platelets were pretreated with thrombin and cultured on fibrinogen micropatterns of varying sizes (median areas: 57 $\mu m^2$ ["large"] and 25 $\mu m^2$ ["small"]) and shapes (circles, ellipses, and triangles). To assess the impact of spatial confinement on the cytoskeleton, phase-contrast images and fluorescence images of fibrinogen (red) and F-actin (green) were acquired from fixed platelets (Figures 1A and S2). Topography and stiffness images of live platelets were obtained using SICM (Figures 1B and S3). Both optical and SICM images reveal that platelets adapted their morphology to the shape of the underlying micropattern. The fibrinogen micropatterns (red) showed high uniformity in area, aspect ratio, and fluorescence intensity (Figure S4). Occasionally, platelets extended beyond the micropattern boundary (particularly on small micropatterns) (Figure 1A, orange arrows) or failed to fully cover the micropattern (especially on large micropatterns) (Figure S2, light-purple arrows). This is also reflected in the ratio of platelet area to micropattern area. On average, this ratio was larger than 100% for platelets on small micropatterns and smaller than 100% for platelets on large micropatterns (Figure S5). For subsequent analysis, only platelets that completely covered their respective fibrinogen micropattern were included.

Parameters quantifying the morphology of confined platelets were extracted from SICM topography images. Across all confinement conditions, the platelet area (Figure 2A) closely matched the area of the

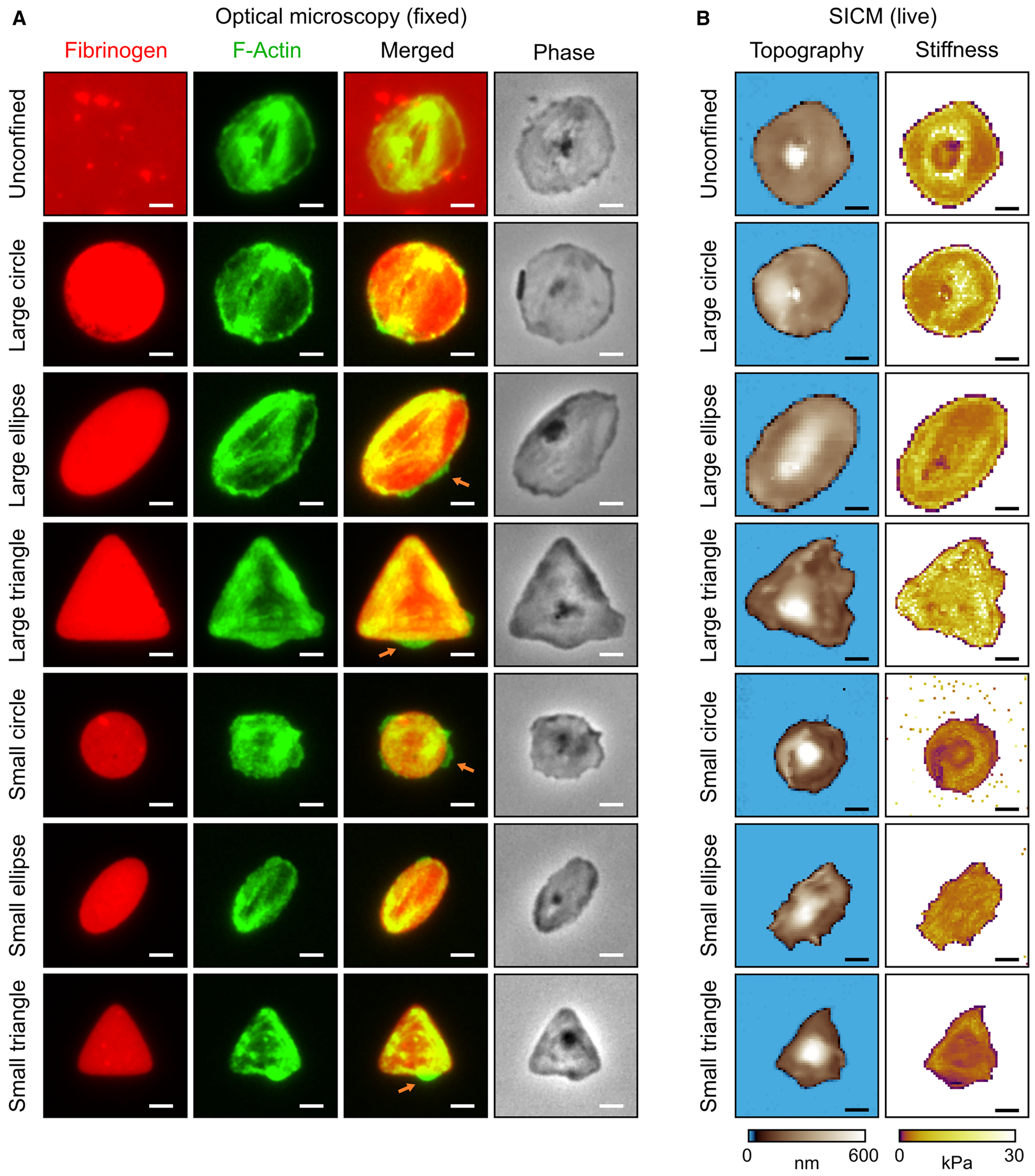


Figure 1 Platelets adapt to spatial confinement. (A) Fibrinogen (red) and F-actin (green) fluorescence with merged and phase-contrast views of platelets on fibrinogen micropatterns. Under confinement, platelets adapted to the imposed shapes and redistributed their F-actin cytoskeleton toward the rim of the pattern. Orange arrows indicate platelets extending beyond micropattern boundaries. Images represent enlarged sections of those shown in Figure S2. Scale bars, 2 μm. (B) Topography and stiffness images of live platelets, obtained via scanning ion conductance microscopy (SICM). Figure S3 provides three-dimensional renderings of platelet topography and height profiles. Scale bars, 2 μm.

corresponding micropattern (Figure S4A, shown as red diamonds in Figure 2A). However, the area of platelets confined to small micropatterns differed significantly from that of unconfined controls. In contrast, the areas of platelets confined to large micropatterns were not significantly different from controls. This indicates that platelets on small micropatterns were constrained by both shape and size, while those on large micropatterns were mainly influenced by shape.

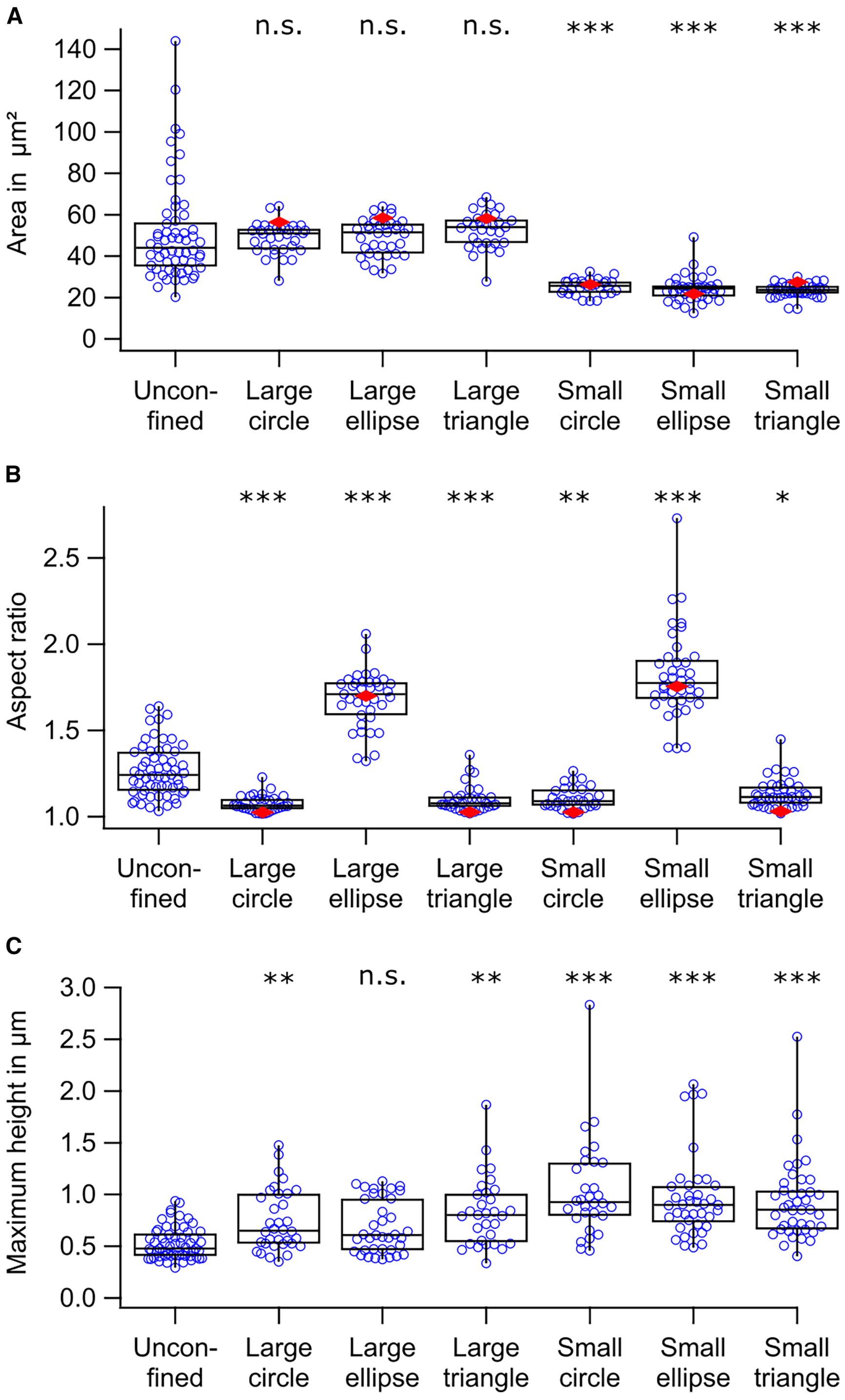


Figure 2 Spatial confinement alters platelet morphology. (A) Platelet area was significantly reduced when confined to small micropatterns compared to unconfined platelets. Median areas of the underlying micropatterns are indicated as red diamonds. (B) Platelet aspect ratio closely reflected the underlying micropattern and was significantly reduced for circle or triangle micropatterns, while significantly increased for ellipse micropatterns, compared to unconfined platelets. Median aspect ratios of micropatterns are shown as red diamonds. (C) Platelet height significantly increased under all confinement conditions, except for large ellipse micropatterns, compared to unconfined platelets. For each condition, $n = 30–59$ platelets from 3–4 donors were analyzed. Significant differences between unconfined condition and confined conditions are indicated by asterisks (*$p < 0.05$, **$p < 0.01$, ***$p < 0.001$; n.s., no significant difference; Dunn's test).

Across all confinement conditions, the aspect ratio closely reflected that of the corresponding micropattern (Figures 2B and S4B). The aspect ratio was significantly reduced on "circle" or "triangle" micropatterns and was significantly increased on "ellipse" micropatterns compared to unconfined platelets (Figure 2B).

The maximum height of platelets (Figure 2C) was significantly increased under spatial confinement across all tested micropatterns, except for the large ellipse micropatterns, when compared to unconfined platelets.

Platelet volume (Figure S6) was significantly reduced in platelets confined to small ellipse or

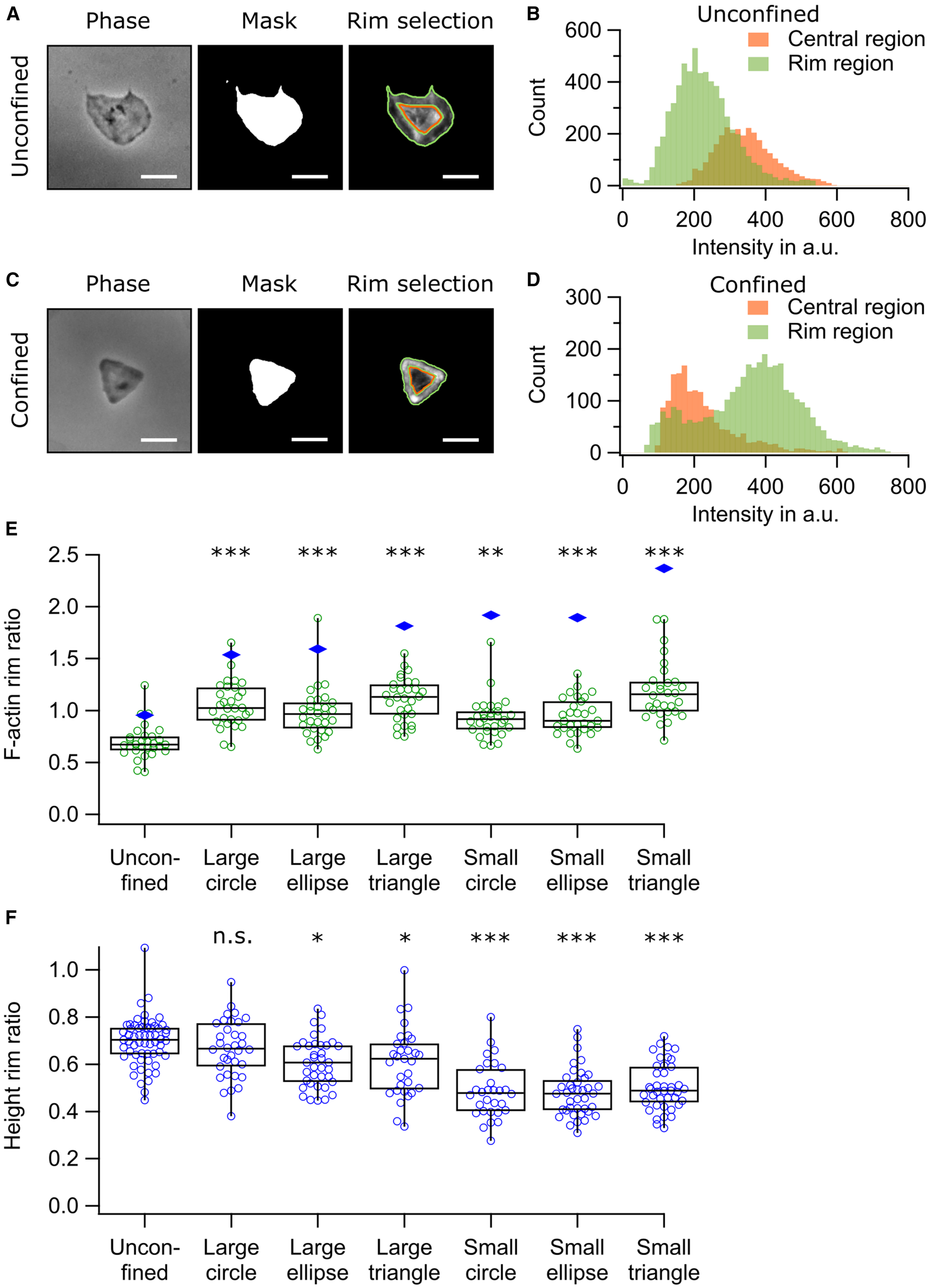


Figure 3 Spatial confinement alters F-actin and height distribution. (A and C) Phase-contrast images (Phase), binary mask of the area (Mask), and F-actin fluorescence images (Rim selection) for unconfined (A) and confined (C) platelets. The rim and central regions are outlined with olive and orange lines, respectively. Scale bars, 5 μm. (B and D) Fluorescence intensity histograms corresponding to (A) and (C), showing F-actin distribution in rim (olive) and central (orange) regions for unconfined (B) and confined (D) platelets. In unconfined platelets,

*(legend continued on next page)*

triangle micropatterns, whereas no significant differences were observed for platelets confined to the other micropatterns when compared to unconfined platelets.

### F-actin redistributed toward the platelet rim under spatial confinement

Fluorescence microscopy revealed that F-actin shifted toward the rim of the micropattern under confinement. Unconfined platelets typically exhibited higher F-actin fluorescence intensity in the central region, whereas confined platelets exhibited similar or lower central intensity compared to the rim (Figures 1A and S2).

To quantify F-actin redistribution, we calculated the "F-actin rim ratio" as the mean F-actin fluorescence intensity at the platelet rim divided by that in the center (see materials and methods) (Figures 3A–3D). This ratio was significantly lower in unconfined platelets compared to those on any micropattern (Figure 3E).

The central region of platelets is typically thicker than the rim, so its F-actin fluorescence intensity reflects a larger volume. For example, a platelet with uniform F-actin would appear brighter in the center than at the rim. To account for this, we calculated the height rim ratio analogous to the F-actin rim ratio (Figure 3F) and introduced a height-corrected volumetric F-actin rim ratio by dividing the median F-actin rim ratio by the median height rim ratio. The volumetric F-actin rim ratio was ≈0.95 in unconfined platelets (Figure 3E, blue diamonds), indicating similar F-actin concentrations in the rim and center. Under spatial confinement, the ratio was between 1.5 and 2.5, indicating an increased F-actin concentration at the rim.

### Platelet stiffness was changed under spatial confinement

Stiffness decreased only on small micropatterns (Figure 4A, markers) compared to unconfined platelets. Large micropatterns showed no significant difference. Stiffness was unaffected by micropattern shape, indicating that size—not shape—determines stiffness.

To assess subcellular stiffness distribution, we calculated the stiffness rim ratio as the geometric mean stiffness at the rim divided by that in the center. Although stiffness was reduced on small micropatterns compared to unconfined platelets (Figure 4A), the stiffness rim ratio remained similar across conditions (Figure 4B), with only small ellipse micropatterns showing a slight but significant reduction.

### Effect of cGMP signaling on platelets under spatial confinement

To assess how activation status affects platelet behavior under spatial confinement, we inhibited activation using 8-Br-cGMP, a membrane-permeable cGMP analog. Platelets were treated with 1 mM 8-Br-cGMP before thrombin activation. Only large and small circle micropatterns were tested, as stiffness was independent of micropattern shape (Figure 4A). Treated platelets adapted well to the shape of the underlying micropattern (Figures 5A and 5B), similar to untreated controls (Figure 1), and exhibited comparable topographical parameters (treated: Figures S7A–S7D; untreated: Figures 2 and S6).

Fluorescence analysis revealed a redistribution of the F-actin cytoskeleton toward the rim of the micropattern (Figure 5A), similar to platelets without 8-Br-cGMP treatment (Figure 1A). F-actin and volumetric rim ratios were comparable between 8-Br-cGMP-treated (Figure 5C) and untreated (Figure 3E) platelets.

A significant reduction in stiffness was observed in 8-Br-cGMP-treated (Figure 5E) compared to untreated (Figure 4A), unconfined platelets ($p \approx 0.0002$; Wilcoxon-Mann-Whitney test). In contrast, platelets treated with 8-Br-cGMP and confined to small or large circle micropatterns showed no significant difference in stiffness compared to the unconfined condition (Figure 5E). These findings indicate that cGMP signaling prevents stiffness reduction in response to spatial confinement.

## DISCUSSION

Building on previous studies for 1D confining microenvironments,[15,16] we demonstrate that platelets

F-actin was predominantly concentrated in the central region compared to the rim. (E) F-actin rim ratio, defined as the mean F-actin fluorescence intensity in the rim region divided by that in the central region, was significantly increased under confinement. Each green marker represents the F-actin rim ratio for an individual platelet ($n = 30$ platelets per condition from three donors). Height-corrected, volumetric F-actin rim ratios were calculated by dividing the median F-actin rim ratio by the median height rim ratio and are shown as blue diamonds. (F) Height rim ratio, defined as the mean height in the rim region divided by that in the central region, was significantly reduced for platelets confined to small micropatterns compared to unconfined platelets. Each dot represents the height rim ratio for an individual platelet ($n =$ 30–59 platelets per condition from 3–4 donors), measured using SICM. Significant differences between unconfined condition and confined conditions are indicated by asterisks (*$p < 0.05$, **$p < 0.01$, ***$p < 0.001$; n.s., no significant difference; Dunn's test).

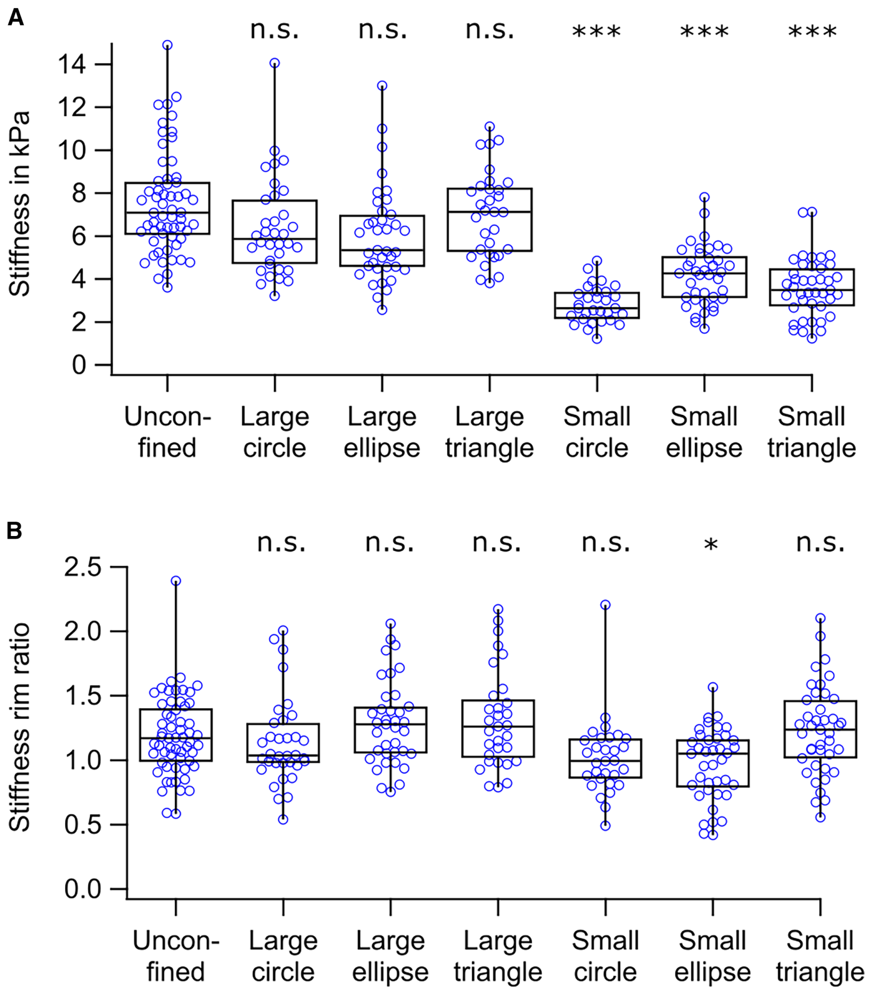


Figure 4 Spatial confinement alters platelet stiffness. (A) Platelet stiffness was significantly reduced when confined to small micropatterns compared to both large micropatterns and unconfined platelets. Each marker represents the median stiffness for an individual platelet. (B) The stiffness rim ratio was comparable across all conditions, indicating a similar subcellular stiffness distribution. The stiffness rim ratio is defined as the geometric mean stiffness of the rim region divided by that of the central region. Each marker represents the stiffness rim ratio for an individual platelet. For each condition, $n = 30–59$ platelets from 3–4 donors were analyzed. Significant differences between unconfined condition and confined conditions are indicated by asterisks (*$p < 0.05$, ***$p < 0.001$; n.s., no significant difference; Dunn's test).

exposed to 2D spatial confinement adapt to varying shapes and sizes, similarly to other cell types.[36–38] Spatial confinement induced morphological changes, consistent with observations in other systems such as RPE1[38] and A7[39] cells.

Interestingly, in contrast to platelets in 1D spatial confinement,[16] platelets in 2D spatial confinement not only adapted their aspect ratio to the underlying micropattern but also adjusted their area and, in some cases, even altered their volume. The median platelet volume was slightly lower for small micropatterns compared to unconfined platelets. However, a significant volume decrease was observed only for small ellipse and triangle micropatterns. One possible explanation is that larger platelets may have been washed away during sample preparation because they cannot adhere efficiently to small adhesive areas. Alternatively, platelets might actively regulate their volume when spreading on micropatterns of different sizes, as has been reported previously for unconfined platelets.[40] To clarify this, future studies could investigate the dynamic behavior of platelets in confinement.

Our observation of F-actin redistribution, as reflected by a significant increase in the F-actin rim ratio in confined platelets compared to unconfined ones, is consistent with the findings of Kita et al. and Sakurai et al., who reported that platelets confined to fibrinogen or collagen micropatterns exhibited the highest F-actin density at the micropattern boundary.[15,17] The platelet rim region seems to be particularly important for thrombin-activated platelets, because they are highly dynamic in this region and actively alter their topography with the involvement of the actin cytoskeleton.[41] In addition to the redistribution of F-actin toward the platelet rim, which we observed for spatially confined platelets, other aspects of F-actin distribution are also likely to be relevant. For example, stress fiber orientation has been found to correlate with the force fields generated by platelets.[42]

The stiffness values measured for unconfined platelets in this study are comparable to those

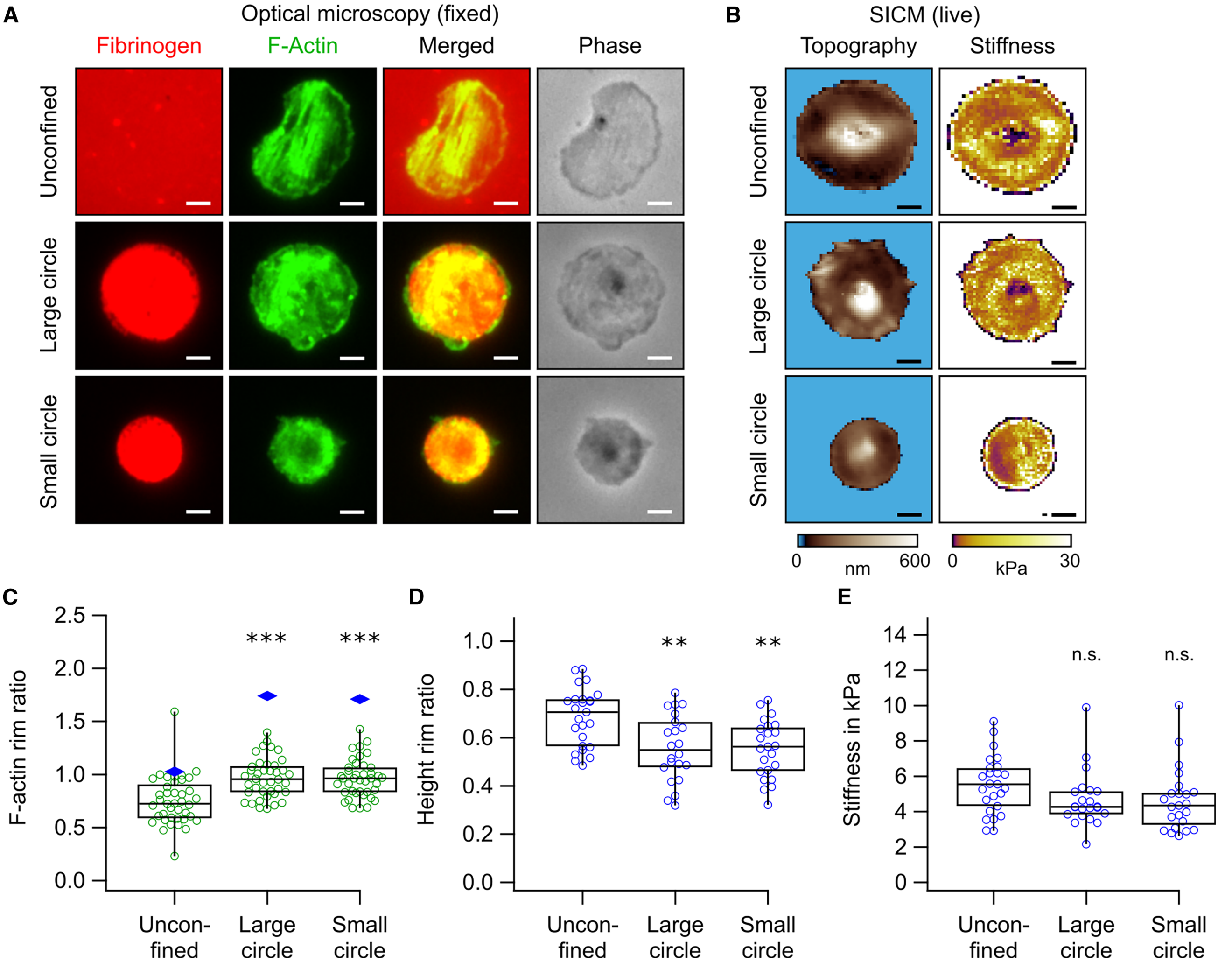


Figure 5 8-Br-cGMP prevents confinement-induced stiffness reduction. (A) Fibrinogen (red) and F-actin (green) fluorescence with merged and phase-contrast views of platelets treated with 1 mM 8-Br-cGMP on micropatterns. Scale bars, 2 μm. (B) Topography and stiffness images of live platelets treated with 1 mM 8-Br-cGMP. Scale bars, 2 μm. (C) F-actin rim ratio was significantly increased in confined platelets. Each green marker represents the F-actin rim ratio for an individual platelet ($n = 40$ platelets per condition from four donors). Height-corrected, volumetric F-actin rim ratios are shown as blue diamonds. (D) Height rim ratio was significantly reduced in confined platelets. Each marker represents the height rim ratio for an individual platelet ($n = 22–25$ platelets per condition from three donors). (E) Stiffness was not significantly affected by spatial confinement. Each marker represents the height rim ratio for an individual platelet ($n = 22–25$ platelets per condition from three donors). Significant differences between unconfined condition and confined conditions are indicated by asterisks (**$p < 0.01$, ***$p < 0.001$; n.s., no significant difference; Dunn's test).

previously reported for unconfined (thrombin-activated) platelets.[20] Reducing the micropattern area significantly decreased platelet stiffness. A softening behavior under confinement has also been observed in other cell types, including A7 cells[39] and tumor cells,[43] which is consistent with our findings. To elucidate whether F-actin contributes to the observed stiffness changes, it would be relevant in future studies to visualize dynamic changes in F-actin, which, for example, occur during platelet spreading,[44] simultaneously with SICM imaging using live actin staining.

In all experiments, platelets were activated with thrombin prior to seeding. This should be taken into account when interpreting the results, as thrombin-induced activation substantially modifies platelet properties; for example, it has been shown to reduce platelet stiffness, likely through actin cytoskeleton remodeling.[20] *In vivo*, thrombin-mediated activation occurs during hemostasis, where fibrinogen plays a key role in platelet aggregation.[45,46] Therefore, the conditions used for our *in vitro* study may mimic aspects of confining microenvironments present during platelet plug formation. Reduced stiffness, e.g., caused by thrombin and/or spatial confinement, could help platelets to squeeze into small cavities when forming platelet plugs, thereby contributing to their stability.

Not only thrombin but also fibrinogen from the micropattern could affect platelet properties. Since the median fibrinogen fluorescence intensity, which reflects fibrinogen density, was similar for all micropatterns, platelets confined to small micropatterns experience less total fibrinogen, which could affect their stiffness. However, platelets also showed a reduction in stiffness in 1D spatial confinement, despite the platelet area (and therefore the total amount of fibrinogen acting on the platelets) not being altered.[16] Therefore, we believe that the stiffness reduction we observed for platelets confined on small micropatterns is not solely due to less total fibrinogen acting on them. The stiffness reduction observed in 1D and 2D spatial confinement may be caused by the same underlying mechanism.

Treatment with 8-Br-cGMP, a membrane-permeable analog of cGMP and inhibitor of platelet activation, reduced the stiffness of unconfined platelets, consistent with previous reports.[22] Under spatial confinement, platelets treated with 8-Br-cGMP exhibited F-actin redistribution comparable to that in untreated platelets but did not show the stiffness reduction observed with decreased micropattern area. This indicates that cGMP-mediated inhibition of platelet activation might partially prevent the biomechanical adaptation of platelets to confining microenvironments.

## DATA AVAILABILITY

The data have been deposited at zenodo.org: https://doi.org/10.5281/zenodo.19112321 and are publicly available as of the article's publication date. Further requests should be addressed to the corresponding author.

## ACKNOWLEDGMENTS

This work was supported by grants from the Deutsche Forschungsgemeinschaft project number 335549539–GRK2381 and project number 374031971–TRR 240 as well as from the Reinhard Frank-Stiftung at the University of Tübingen. A.B. is supported by an Add-on Fellowship of the Joachim Herz Foundation. The authors acknowledge support from the High Performance and Cloud Computing Group at the Zentrum für Datenverarbeitung of the University of Tübingen, the state of Baden-Württemberg through bwHPC, and the German Research Foundation through grant no. INST 37/935-1 FUGG. We acknowledge support from the Open Access Publication Fund of the University of Tübingen.

## AUTHOR CONTRIBUTIONS

A.B., V.G., and T.E.S. designed the study; A.B. and V.G. performed the experiments; A.B., V.G., and T.E.S. analyzed data and wrote the manuscript; and A.B., V.G., and T.E.S. interpreted data, discussed results, and revised the manuscript. All authors have read and agreed to the final version of the manuscript.

## DECLARATION OF INTERESTS

The authors declare no competing interests.

## SUPPLEMENTAL INFORMATION

Supplemental information can be found online at https://doi.org/10.1016/j.bpr.2026.100261.

## REFERENCES

1. Nurden, A. T. 2011. Platelets, inflammation and tissue regeneration. *Thromb. Haemost.* 105:S13–S33.
2. Storey, R., and M. Thomas. 2017. The role of platelets in inflammation. *Thromb. Haemost.* 114:449–458.
3. Broos, K., H. B. Feys, …, H. Deckmyn. 2011. Platelets at work in primary hemostasis. *Blood Rev.* 25:155–167.
4. Ghoshal, K., and M. Bhattacharyya. 2014. Overview of Platelet Physiology: Its Hemostatic and Nonhemostatic Role in Disease Pathogenesis. *Sci. World J.* 2014:781857.
5. Stalker, T. J., D. K. Newman, …, L. F. Brass. 2012. Platelet signaling. *In* Antiplatelet Agents. Handbook of Experimental Pharmacology, *210*. P. Gresele, G. V. R. Born, C. Patrono, and C. P. Page, eds. Springer, pp. 59–85.
6. Gear, A. R. 1984. Rapid platelet morphological changes visualized by scanning-electron microscopy: kinetics derived from a quenched-flow approach. *Br. J. Haematol.* 56:387–398.
7. Posch, S., I. Neundlinger, …, A. Ebner. 2013. Activation induced morphological changes and integrin alphaIIbbeta3 activity of living platelets. *Methods.* 60:179–185.
8. Ruggeri, Z. M. 2002. Platelets in atherothrombosis. *Nat. Med.* 8:1227–1234.
9. Andrews, R. K., E. E. Gardiner, …, M. C. Berndt. 2004. Platelet Interactions in Thrombosis. *IUBMB Life.* 56:13–18.
10. Link, R., K. Weißenbruch, …, U. S. Schwarz. 2023. Cell Shape and Forces in Elastic and Structured Environments: From Single Cells to Organoids. *Adv. Funct. Mater.* 34:2302145.
11. Chen, C. S., M. Mrksich, …, D. E. Ingber. 1997. Geometric control of cell life and death. *Science.* 276:1425–1428.
12. Balzer, E. M., Z. Tong, …, K. Konstantopoulos. 2012. Physical confinement alters tumor cell adhesion and migration phenotypes. *FASEB J.* 26:4045–4056.
13. Lavi, I., N. Meunier, …, J. Casademunt. 2020. Motility and morphodynamics of confined cells. *Phys. Rev. E.* 101:022404.
14. Mosier, J. A., E. D. Fabiano, …, C. A. Reinhart-King. 2023. Confinement primes cells for faster migration by polarizing active mitochondria. *Nanoscale Adv.* 6:209–220.
15. Kita, A., Y. Sakurai, …, W. A. Lam. 2011. Microenvironmental Geometry Guides Platelet Adhesion and Spreading: A Quantitative Analysis at the Single Cell Level. *PLoS One.* 6:e26437.
16. Rodríguez, J. G. 2025. Spatial confinement alters morphology, spreading dynamics, and mechanics of adherent platelets. *Biophysical Reports.* 5:100222.
17. Sakurai, Y., J. L. Fitch-Tewfik, …, W. A. Lam. 2015. Platelet geometry sensing spatially regulates α-granule secretion to enable matrix self-deposition. *Blood.* 126:531–538.
18. Liu, X., Y. Li, …, Y. Zhang. 2015. Use of non-contact hopping probe ion conductance microscopy to investigate dynamic morphology of live platelets. *Platelets.* 26:480–485.
19. von Eysmondt, H., J. Seifert, …, T. E. Schäffer. 2025. Mechanosensing alters platelet migration. *Acta Biomater.* 196:213–221.
20. Rheinlaender, J., S. Vogel, …, T. E. Schäffer. 2015. Imaging the elastic modulus of human platelets during thrombininduced

activation using scanning ion conductance microscopy. *Thromb. Haemost.* 113:305–311.

21. Seifert, J., J. Rheinlaender, ..., T. E. Schäffer. 2022. Mechanics of migrating platelets investigated with scanning ion conductance microscopy. *Nanoscale.* 14:8192–8199.
22. Balmes, A., J. G. Rodríguez, ..., T. E. Schäffer. 2024. Role of the NO-GC/cGMP signaling pathway in platelet biomechanics. *Platelets.* 35:2313359.
23. Schaale, D., Z. Laspa, ..., M. Gawaz. 2024. Hemin promotes platelet activation and plasma membrane disintegration regulated by the subtilisin-like proprotein convertase furin. *FASEB J.* 38:e70155.
24. Schäffer, T. E. 2013. Nanomechanics of molecules and living cells with scanning ion conductance microscopy. *Anal. Chem.* 85:6988–6994.
25. Zhu, C., K. Huang, ..., L. A. Baker. 2021. Scanning ion conductance microscopy. *Chem. Rev.* 121:11726–11768.
26. Fritz, M., M. Radmacher, and H. E. Gaub. 1993. In Vitro Activation of Human Platelets Triggered and Probed by Atomic Force Microscopy. *Exp. Cell Res.* 205:187–190.
27. Hofmann, F. 2020. The cGMP system: components and function. *Biol. Chem.* 401:447–469.
28. Emerson, M., S. Momi, ..., P. Gresele. 1999. Endogenous nitric oxide acts as a natural antithrombotic agent in vivo by inhibiting platelet aggregation in the pulmonary vasculature. *Thromb. Haemost.* 81:961–966.
29. Antl, M., M. L. von Brühl, ..., J. Schlossmann. 2007. IRAG mediates NO/cGMP-dependent inhibition of platelet aggregation and thrombus formation. *Blood.* 109:552–559.
30. Gambaryan, S. 2022. The Role of NO/sGC/cGMP/PKG Signaling Pathway in Regulation of Platelet Function. *Cells.* 11:3704.
31. Seifert, J., J. Rheinlaender, ..., T. E. Schäffer. 2015. Comparison of Atomic Force Microscopy and Scanning Ion Conductance Microscopy for Live Cell Imaging. *Langmuir.* 31:6807–6813.
32. Krutzke, K., J. Seifert, ..., T. E. Schäffer. 2024. Volume Regulation and Nonosmotic Volume of Individual Human Platelets Quantified by High-Speed Scanning Ion Conductance Microscopy. *Thromb. Haemost.* 125:340.
33. Rheinlaender, J., and T. E. Schäffer. 2013. Mapping the mechanical stiffness of live cells with the scanning ion conductance microscope. *Soft Matter.* 9:3230–3236.
34. Rheinlaender, J., and T. E. Schäffer. 2020. The effect of finite sample thickness in scanning ion conductance microscopy stiffness measurements. *Appl. Phys. Lett.* 117:113701.
35. Seifert, J., H. von Eysmondt, ..., T. E. Schäffer. 2021. Effect of Oxidized LDL on Platelet Shape, Spreading, and Migration Investigated with Deep Learning Platelet Morphometry. *Cells.* 10:2932.
36. Versaevel, M., T. Grevesse, and S. Gabriele. 2012. Spatial coordination between cell and nuclear shape within micropatterned endothelial cells. *Nat. Commun.* 3:671.
37. Grevesse, T., M. Versaevel, ..., S. Gabriele. 2013. A simple route to functionalize polyacrylamide hydrogels for the independent tuning of mechanotransduction cues. *Lab Chip.* 13:777–780.
38. Rigato, A., F. Rico, ..., S. Scheuring. 2015. Atomic Force Microscopy Mechanical Mapping of Micropatterned Cells Shows Adhesion Geometry-Dependent Mechanical Response on Local and Global Scales. *ACS Nano.* 9:5846–5856.
39. Guo, M., A. F. Pegoraro, ..., D. A. Weitz. 2017. Cell volume change through water efflux impacts cell stiffness and stem cell fate. *Proc. Natl. Acad. Sci. USA.* 114:E8618–E8627.
40. Krutzke, K., J. Seifert, ..., J. Rheinlaender. 2026. Platelet stiffness correlates inversely with platelet volume during expansion and compression. *Soft Matter.* 22:578–588.
41. Seifert, J., J. Rheinlaender, ..., T. E. Schäffer. 2017. Thrombin-induced cytoskeleton dynamics in spread human platelets observed with fast scanning ion conductance microscopy. *Sci. Rep.* 7:4810.
42. Zelená, A., J. Blumberg, ..., S. Köster. 2023. Force generation in human blood platelets by filamentous actomyosin structures. *Biophys. J.* 122:3340–3353.
43. Rianna, C., M. Radmacher, and S. Kumar. 2020. Direct evidence that tumor cells soften when navigating confined spaces. *Mol. Biol. Cell.* 31:1726–1734.
44. Paknikar, A. K., B. Eltzner, and S. Köster. 2019. Direct characterization of cytoskeletal reorganization during blood platelet spreading. *Prog. Biophys. Mol. Biol.* 144:166–176.
45. Brass, L. F. 2003. Thrombin and platelet activation. *Chest.* 124:18s–25s.
46. Ruggeri, Z. M. 2009. Platelet adhesion under flow. *Microcirculation.* 16:58–83.

Biophysical Reports, Volume *6*

# Supplemental information

# Effect of two-dimensional spatial confinement on platelet mechanics

**Aylin Balmes, Vincent Gidlund, and Tilman E. Schäffer**

# Supplemental Information

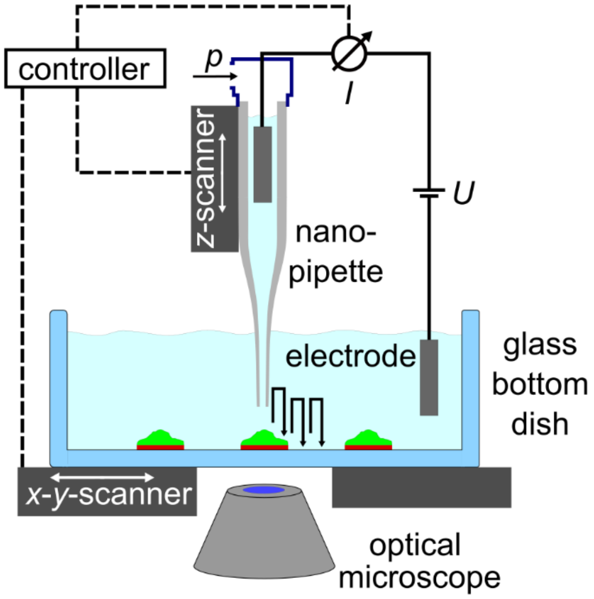


**Figure S1: Principle of scanning ion conductance microscopy (SICM).** A voltage (*U*) applied between two electrodes - one in the electrolyte-filled nanopipette and the other in the bath - induces an ion current (*I*) that depends on the pipette-sample distance. The topography of the sample is imaged by scanning it using an *x*-*y*-scanner. At every *x*-*y*-position, the nanopipette is vertically approached toward the sample until the ion current drops to a predetermined threshold, at which point the corresponding *z*-position of the nanopipette is recorded. The recorded *z*-positions make up the topography image. For stiffness imaging, a pressure (*p*) is applied to the top end of the nanopipette. When approaching the pipette to a soft sample, it is deformed by the outward flow from the pipette, resulting in a shallower slope of the ion current vs. distance curve than for a stiff sample. Stiffness is derived from this slope [1].

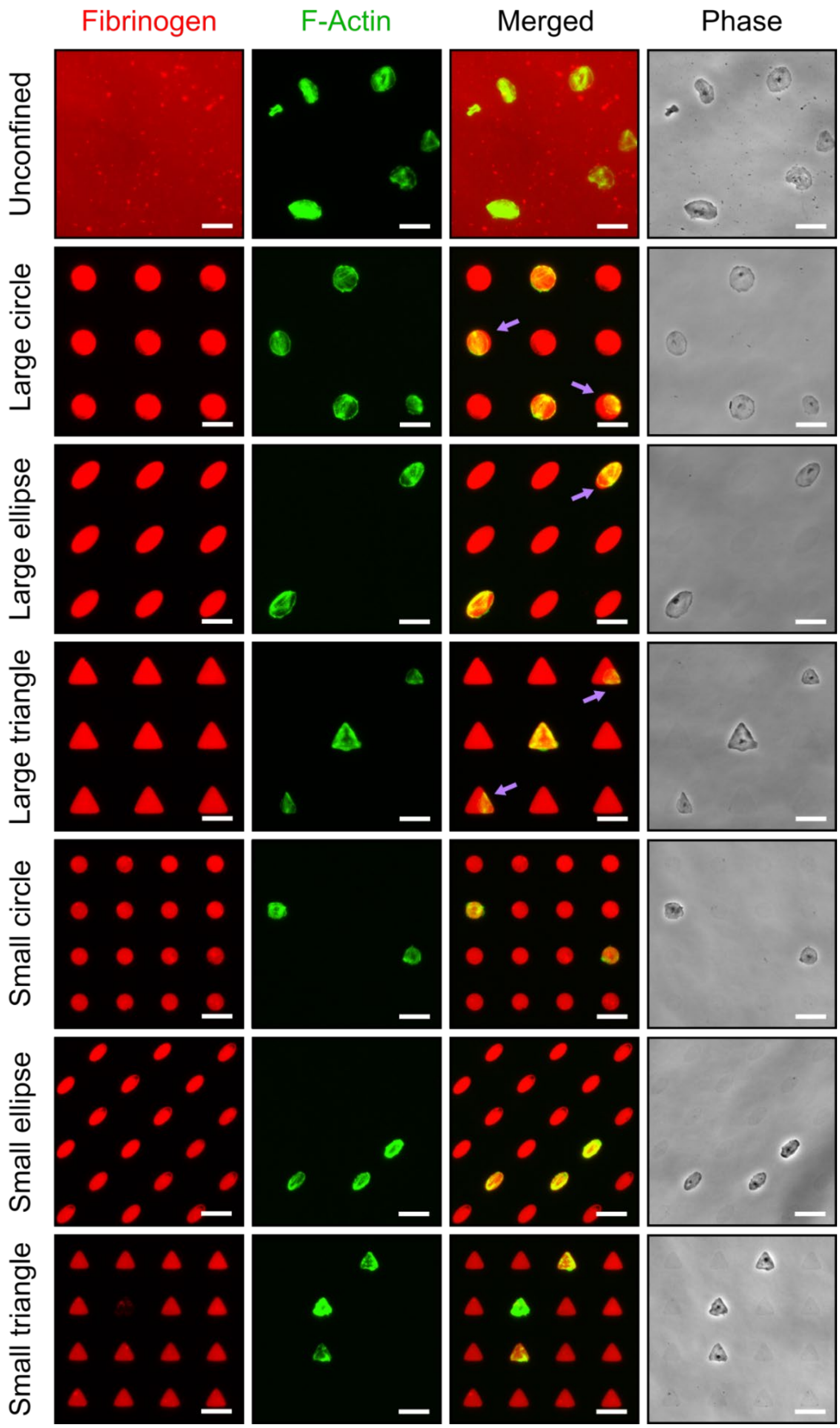


**Figure S2: Overview of fibrinogen micropatterns and spatially confined platelets.** Fibrinogen (red) and F-actin (green) fluorescence with merged and phase contrast views of platelets on fibrinogen micropatterns of varying shape and size. Light purple arrows indicate platelets that do not fully cover the micropatterns. Scale bars: 10 µm.

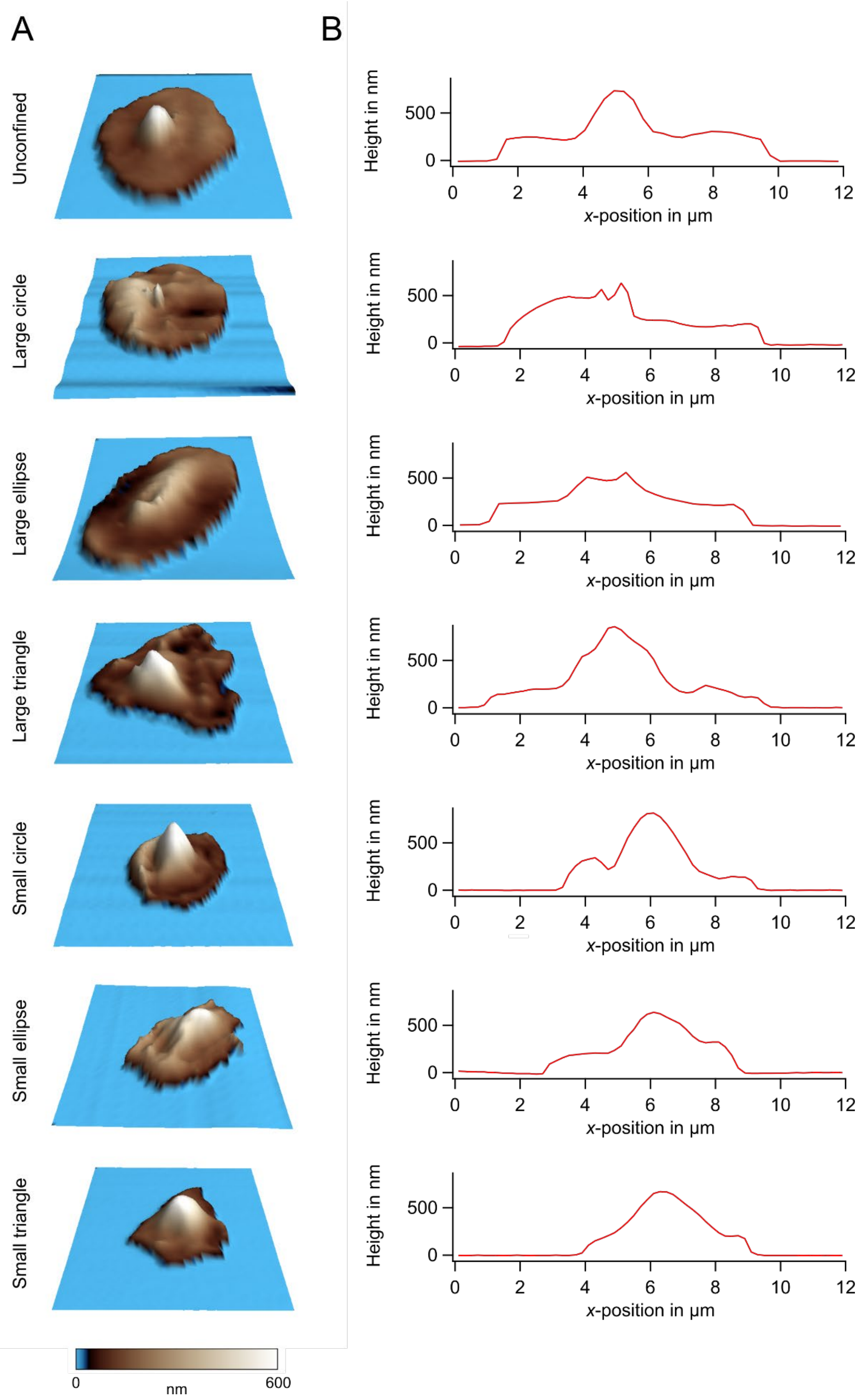


**Figure S3: Visualization of three-dimensional platelet topography.** (**A**) Three-dimensional renderings of platelet surface topography. The *x*-*y*-image size is 12 × 12 µm². (**B**) Respective height profiles in the horizontal (*x*) direction, taken through the point of maximum height.

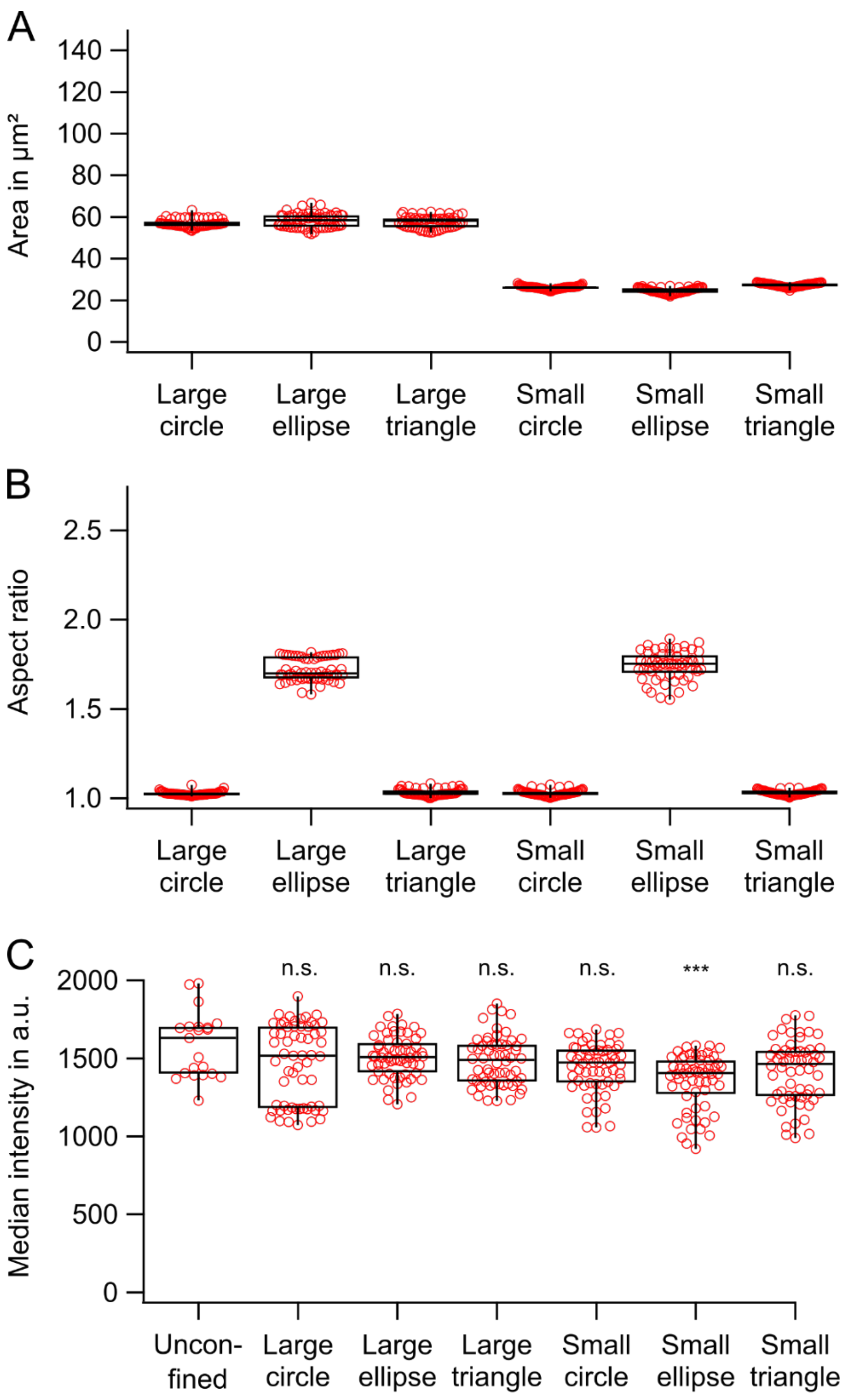


**Figure S4: Fibrinogen micropatterns are highly uniform in shape and fluorescence.** (**A**) Area and (**B**) aspect ratio are highly uniform. (**C**) The median fibrinogen fluorescence intensity of the micropatterns was not significantly different compared to flat printings (unconfined), except for the small ellipse micropatterns, where a slight, but significant decrease was observed (*n*=60 micropatterns, *n*=21 flat printings; Dunn's test).

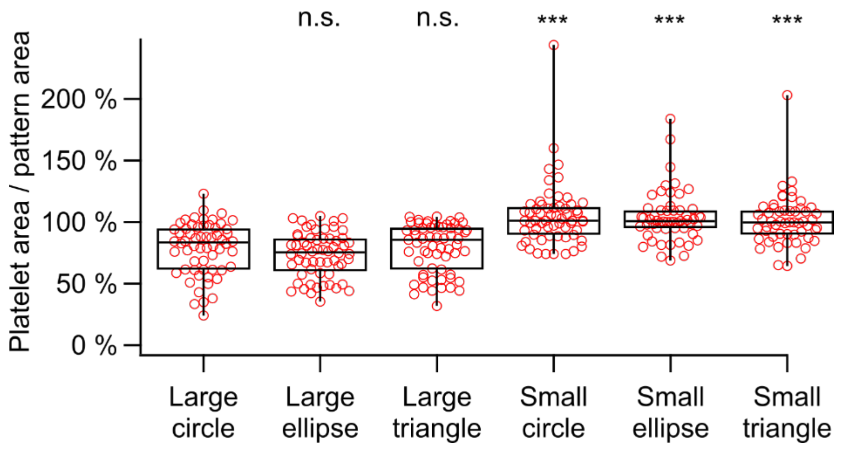


**Figure S5: Ratios of platelet area to underlying pattern area are significantly higher for small patterns compared to large patterns**. For each condition, $n$=54–62 platelets from 3–4 donors were analyzed. Significant differences between conditions are indicated by asterisks (*: $p < 0.05$; **: $p < 0.01$; ***: $p < 0.001$; n.s. indicates no significant difference; Dunn's test).

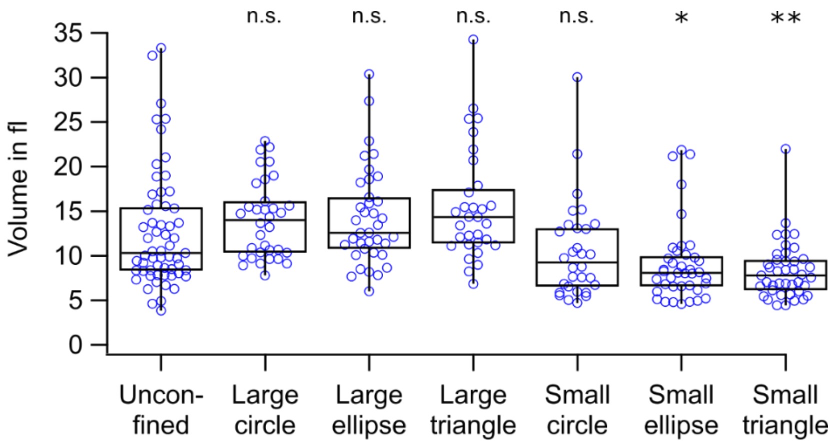


**Figure S6: Platelet volume under spatial confinement.** Volume was significantly reduced on small ellipse and small triangle micropatterns, compared to unconfined platelets ($n$=30–59 platelets per condition from 3–4 donors). Significant differences between unconfined condition and confined conditions are indicated by asterisks (*: $p < 0.05$; **: $p < 0.01$; ***: $p < 0.001$; n.s. indicates no significant difference; Dunn's test).

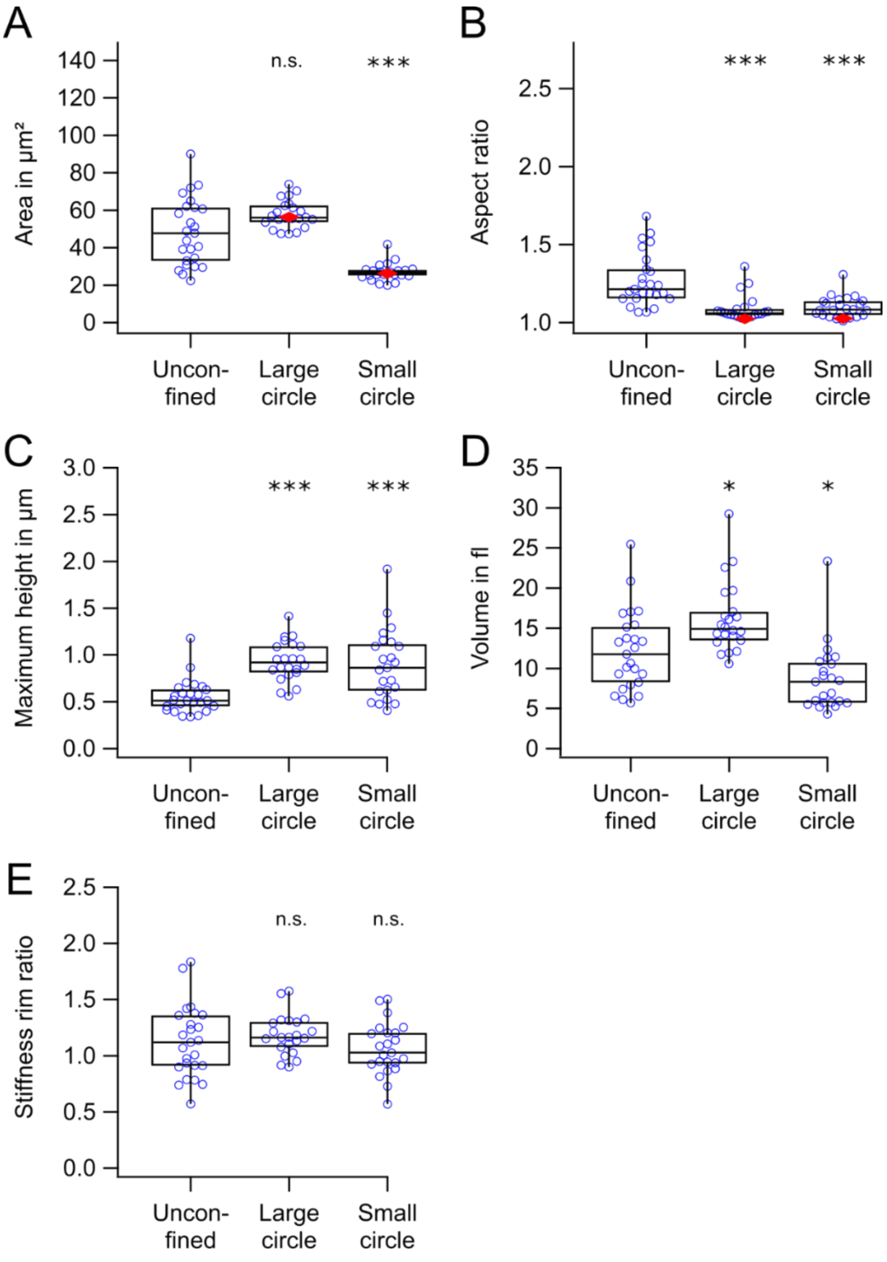


**Figure S7: Topographical parameters and stiffness rim ratio of platelets treated with 8-Br-cGMP.** (**A**) Platelet area was significantly reduced for platelets confined to small circle micropatterns, compared to unconfined platelets. Median areas of microcontact printed micropatterns are shown as red diamonds. (**B**) Platelet aspect ratio was significantly reduced for platelets confined to large or small circle micropatterns, compared to unconfined platelets. Median aspect ratios of microcontact-printed micropatterns are shown as red diamonds. (**C**) Platelet maximum height was significantly increased for platelets confined to large or small circle micropatterns, compared to unconfined platelets. (**D**) Platelet volume was significantly increased or reduced when confined to large or small circle micropatterns, respectively, compared to unconfined platelets. (**E**) Stiffness rim ratio was not significantly altered by spatial confinement. Each marker represents an individual platelet. Per condition, 22–25 platelets from 3 donors were measured. Significant differences between unconfined condition and confined conditions are indicated by asterisks (*: $p < 0.05$; **: $p < 0.01$; ***: $p < 0.001$; n.s. indicates no significant difference; Dunn's test).

## References

1. Rheinlaender, J. and T.E. Schäffer, *Mapping the mechanical stiffness of live cells with the scanning ion conductance microscope.* Soft Matter, 2013. **9**(12): p. 3230–3236.
